# 

**Published:** 1915-11-27

**Authors:** 


### CONTENTS.

PAGE

**Unqualified Practice** ... ... ... ... ... ... 173

**Hughlings Jackson** ... ... ... ... ... 174

**Hospital and Institutional News** ... ... ... 175

Our Novel Christmas Number—Readers! Lend a Hand—The Inevitable Happens—The British Hospitals Association—Some Points Worthy of Attention—Should a Hospital Build in War Time?—A Typical German Slander—The South Coast Military Hospitals—Workers and the Maintenance of Infirmaries—Dr. Brend and the Limoges Hospital—Poor-Law Officials and Enlistment—Poor-Law Amalgamation in Shropshire—New Medical Benefit Regulations—An Important Case—A Nice Point—The Glasgow Women's Private Hospital—The Women's Pay Hospital at Dundee—The Rights of Asylum Attendants—The London Jewish Hospital—Death of Dr. Bastian—Working Men's Support in the Midlands—Extortion and Charity Fêtes—Industrial Schools *versus* Home Life—Ranji's Gift to Leeds Infirmary—Nottingham Saturday Fund and the Wounded—More Accommodation at Edmonton for Wounded—Poplar Hospital's Diamond Jubilee—This Week's Drug Market.

**Some Medical Lessons of the War** ... ... 181

The Control of Camp Diseases.

**A Medical Causerie** ... ... ... ... ... 183

PAGE

**Round the World** ... ... ... ... ... ... 184

Fellow-Workers and the Roll of Honour.

**King Edward's Hospital Fund** ... ... ... 185

The Statistical Report for 1914.

**The Blind Soldier** ... ... ... ... ... 186

**Nurses' Accommodation at Glasgow Hospitals** 187

An Educative Contrast and a Revelation.

**The Home Production of Food** ... ... ... 187

**Phthisis in the Boot and Shoe Industry** ... 188

Medical Research Committee (First Report).

**The British Hospitals Association** ... ... ... 189

Mr. Wade Deacon's Address.

**Parliament and the Profession** ... ... ... 190

**Institutional Pharmacists and Recruiting** ... 190

**The Editor's Letter-Box** ... ... ... ... 191

"A British Hospital at Limoges."

**The Northern Association of Hospital Secretaries** ... ... ... ... ... ... ... 191

**Passing Events and Topics** ... ... ... ... 192

**Gifts and Bequests** ... ... ... ... ... 192

**A Curious Story** ... ... ... ... ... 192

**Obituary** ... ... ... ... ... ... ... 192

**The Institutional Worker** ... ... (Suppl.) 1

Palatable Fare Without Meat.

Dispensing Pupils: Answer to October Question.

Enquiries and Answers:

